# TAVI for Pure Non-calcified Aortic Regurgitation Using a Self-Expandable Transcatheter Heart Valve

**DOI:** 10.3389/fcvm.2021.743579

**Published:** 2022-01-25

**Authors:** Yvonne Schneeberger, Moritz Seiffert, Andreas Schaefer, Oliver D. Bhadra, Niklas Schofer, Simon Pecha, Dirk Westermann, Stefan Blankenberg, Hermann Reichenspurner, Lenard Conradi

**Affiliations:** ^1^Department of Cardiovascular Surgery, University Heart and Vascular Center Hamburg, Hamburg, Germany; ^2^Department of Cardiology, University Heart and Vascular Center Hamburg, Hamburg, Germany

**Keywords:** transcatheter aortic valve implantation, aortic valve, aortic regurgitation, self- expanding, transcatheter heart valve

## Abstract

**Objectives:** Transcatheter aortic valve implantation (TAVI) is routinely performed in patients with severe aortic stenosis (AS). For patients with pure non-calcified aortic regurgitation (AR) who are not suitable for open heart surgery no clear recommendations exist and use of TAVI has been largely off-label. We herein report a series of patients treated with the self-expandable AcurateNeo and Neo2 (Boston Scientific Co., Marlborough, MS, USA) transcatheter heart valve (THV) for pure AR.

**Methods:** Between 05/2017 and 03/2021, 9 patients (88.8% female, 74.4 ± 7.1 years, logEuroSCORE II 5.5 ± 3.6%, STS PROM 6.2 ± 3.0%) received transfemoral (TF) TAVI for pure non-calcified AR following an adjusted valve sizing algorithm. Data were retrospectively analyzed according to updated Valve Academic Research Consortium (VARC-2) definitions.

**Results:** Device success was 100%. Early safety was 77.7% (7/10), due to two (22.2%) cases of acute kidney injury. Thirty-day mortality was 0%, in seven (77.7%) patients no or trace paravalvular leakage (PVL) was seen and mild PVL in two (22.2%) patients at 30-day follow-up. No permanent pacemaker (PPM) was required during 30-day follow-up.

**Conclusion:** In this series of selected patients using the Acurate neo THV for pure non-calcified AR, safety and efficacy were demonstrated. Thirty-day mortality as well as PPM implantation and PVL rates showed excellent results in this high-risk patient cohort. These results will have to be confirmed in larger patient cohorts.

## Introduction

Transcatheter aortic valve implantation (TAVI) is routinely performed in patients with severe aortic stenosis (AS) at intermediate or high risk for surgical aortic valve replacement (SAVR), when anatomical conditions for an interventional approach are adequate (1–3). Correspondingly, TAVI has been incorporated in international guidelines (4, 5). While extension of TAVI to low risk patients remains controversial, mainly due to a higher risk of postprocedural permanent pacemaker (PPM) implantation, residual paravalvular leakage (PVL) and lack of long-term durability data as shown in registry analyses (6–8), evolution of transcatheter heart valves (THV) and corresponding delivery systems is continuing. Besides liberalization of TAVI indications, off-label use of THV for varying aortic valve diseases has been described, with broader clinical application in TAVI for pure non-calcified AR. Traditionally, pure AR is considered a contraindication for TAVI, since absence of aortic valve calcification can lead to insufficient anchoring of the stent frame with possible consecutive valve embolization or relevant PVL (9). However, patients with AR and high comorbidity burden may not be eligible for SAVR and the only THV certificated for AR due to a unique anchoring mechanism (JenaValve) has just very recently been commercially approved and is clinically not widely available (10). Patients with untreated severe AR and a left ventricular ejection fraction of < 30% have an annular mortality of up to 20% and only few of these patients undergo SAVR. Since AR prevalence increases with age, an increasing number of patients with AR in need for TAVI can be anticipated (11). The most frequently utilized THV for treatment of AR are reported to be the self-expandable (SE) CoreValve EvolutR (Medtronic PLC., Minneapolis, MN, US) (12), the SE Acurate neo (Boston Scientific Co., Marlborough, MS, US) (13) and balloon-expandable (BE) THV of the Sapien family (Edwards Lifesciences Inc., Irvine, CA, US) (14). Since particular design features of the Acurate neo THV (distal stabilization arches and upper/lower stent crown inner/outer pericardium skirts) have the potential to protect against valve embolization and residual PVL in non-calcified aortic valves it has become our default THV for treatment of pure AR in patients with a prohibitive risk for SAVR. We herein present our experience with this THV platform for treatment of pure AR with a special emphasis on preprocedural planning and intraprocedural considerations.

## Materials and Methods

### Patients

Between 05/2017 and 03/2021, 9 consecutive patients received transfemoral (TF) TAVI using the Acurate neo and neo2 THV for pure non-calcified AR following an adjusted valve sizing algorithm. Assessment of prohibitive risk for SAVR [3/9 patients with previous cardiac surgery, 4/9 due to age and comorbidities, 1/9 due to previous left ventricular assist device (LVAD) implantation and 1/9 due to malignant disease] and allocation to TAVI followed current international recommendations (4, 5) after consensus of the local dedicated heart team. Written informed consent was obtained from all patients. The study was approved by the local institutional review board.

### Diagnostic Work-Up, Study Procedure, and Valve Sizing Algorithm

The preprocedural diagnostic work-up followed institutional standards and was described before (15): By routine, all patients received preoperative transthoracic and/or transesophageal echocardiography (TEE), a contrast-enhanced, electrocardiogram-gated MSCT for calculation for native aortic annulus dimensions and determination of adequate THV size as well as assessment of aortic root anatomy and morphology with the 3mensio Medical Imaging Software (3mensio, Medical Imaging, Bilthoven, Netherlands). Valve sizing followed an adjusted algorithm proposed by Kim et al. for aortic valve stenosis (16) with additional oversizing equivalent to 10.7 ± 2.7% of the THV diameter when compared to the native annulus diameter in this series (see Table 1). As a crude measure, an effective perimeter-derived annular diameter of 25.5 mm was considered the absolute technical maximum even though secondary measures such as annular eccentricity and diameter of the left ventricular outflow tract were also considered and may have contraindicated procedures below this value.

**Table 1:** Transcatheter heart valve sizes and corresponding oversizing values.

**Pat. No**	**Perimeter (mm)**	**Area (mm^**2**^)**	**Perimeter derived diameter (mm)**	**Area-derived diameter (mm)**	**Diameter min (mm)**	**Diameter max (mm)**	**Eccentricity index**	**LVOT diameter (mm)**	**SOV diameter (mm)**	**STJ diameter (mm)**	**STJ height (mm)**	**Ascending aorta diameter (mm)**	**THV size**	Cover index^*^
1	78.1	476.7	24.9	24.6	22.6	26.7	0.2	26.2	43.2	32.2	34.9	32.0	L	7.8
2	66.2	337.7	21.1	20.7	18.1	23.7	0.2	24.6	23.3	19.7	19.7	20.9	S	8.3
3	68.8	370.0	21.9	21.7	19.4	23.7	0.2	27.4	33.9	33.8	25.5	35.4	M	12.4
4	74.9	434.4	23.8	23.5	20.8	26.9	0.2	28.1	36.8	31.4	25.6	33.9	L	11.9
5	71.7	404.1	22.8	22.7	20.9	24.5	0.1	25.9	36.2	32.5	23.7	35.2	M	8.8
6	70.5	390.3	22.4	22.3	20.6	24.1	0.1	29.5	30.6	27.9	20.3	32.7	M	10.4
7	76.9	460.6	24.5	24.2	21.4	26.6	0.2	28.8	39.8	35.6	24.1	42.5	L	9.3
8	73.2	406.7	23.3	22.8	19.7	26.9	0.3	27.0	35.5	27.9	24.0	33.6	L	13.7
9	66.9	344.5	21.3	20.9	18.1	23.0	0.2	22.9	30.6	26.7	23.5	30.7	M	14.8

*LVOT, Left ventricular outflow tract; SOV, Sinus of valsalva; STJ, Sinotubular junction; THV, Transcatheter heart valve*.

* ([nominal THV diameter-measured diameter]/nominal THV diameter)*100.

First line approach for all procedures was local anesthesia and/or analgosedation. All procedures were performed in a specially equipped hybrid operating suite by a dedicated team of cardiologists, cardiac surgeons and anesthesiologists. The first step of THV deployment was conducted on the beating heart using fast pacing while, during the second deployment step, rapid ventricular pacing (RVP) was used to ensure stable THV positioning. In the LVAD patient, the guidewire was placed adjacent to the LVAD inflow by fluoroscopy and TEE guidance and LVAD flow was minimized during THV deployment to avoid ventricular migration. THV function was assessed by invasive measurements of hemodynamics, aortic root angiography, and TTE.

### Transcatheter Heart Valve

**Figure 1:**
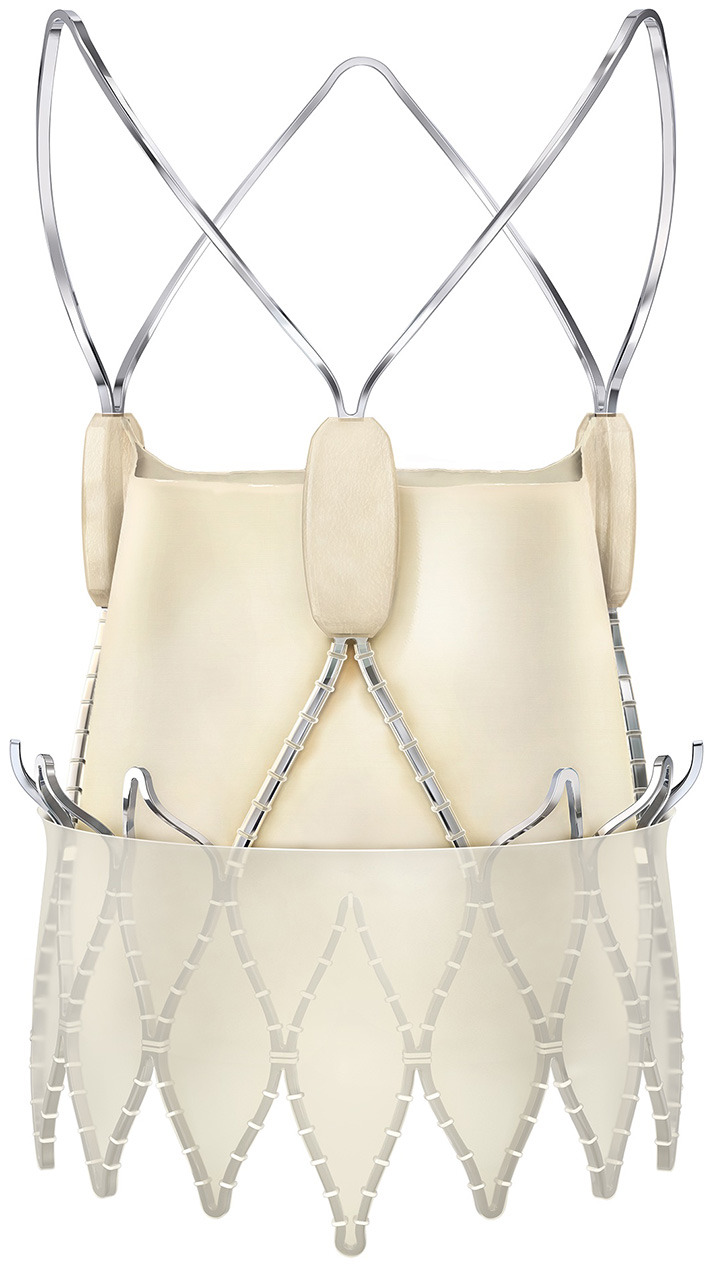
The self-expandable Acurate neo 2 THV. Latest generation self-expandable transcatheter heart valve consisting of a Nitinol stent carrying a porcine pericardial bioprosthesis in supra-annular position, deployment is carried out in a top-down fashion with opening of the upper crown and stabilization arches as first step for stable positioning and coaxial alignement and deployment of the lower valve stent for anchoring in the aortic annulus. Image provided courtesy of Boston Scientific. ©2021 Boston Scientific Corporation or its affiliates. All rights reserved. The usage of Acurate neo2™ in aortic insufficiency is off-label use.

The Boston Scientific Acurate neo (Boston Scientific, Marlborough, MA, USA) THV has a SE nitinol frame carrying porcine pericardial leaflets in a supraannular position (Figure 1). The most important difference to other self-expanding platforms is the top-down deployment with minimal protrusion of the stent toward the left ventricular outflow tract. In addition, supraannular leaflet function provides very low gradients even in small anatomies (17) and the pericardial skirt in the new generation neo 2 design seals effectively against PVL. The transfemoral delivery system has a 18Fr outer diameter shaft. In detail, first the upper crown is opened, which guarantees stable positioning and supraannular anchoring of the valve. Then the flexible stabilization arches are opened, responsible for the self-aligning properties of the valve, thereby ensuring coaxial alignment. Finally, in step two the lower crown is deployed anchoring the device inside the annulus. The THV is available in three sizes for aortic annulus sizes from 20.0 to 26.3 mm and are labeled small (S: for aortic annulus sizes 20.0–22.4 mm), medium (M: for aortic annulus sizes 22.5–24.3 mm) and large (L: for aortic annulus sizes 24.4–26.3 mm) (16, 18) in AS patients.

### Statistics

Baseline, intraprocedural and acute follow-up data up to 30 days were retrospectively collected and entered into a standardized database and analyzed. Clinical endpoints were adjudicated in accordance with the updated standardized VARC-2 definitions (19). Data are presented as absolute numbers and percentages for categorical variables and mean values and standard deviation for continuous variables.

## Results

### Baseline Demographics

All 9 consecutive patients (88.8% female, 74.4 ± 7.1 years) demonstrated an increased risk profile as reflected by common risk stratification tools (EuroSCORE II 5.5 ± 3.6%, STS PROM 6.2 ± 3.0%). Patients presented with a high comorbidity burden, including 3/9 (33.3%) with previous sternotomy, 3/9 (33.3%) patients with concomitant coronary artery disease, and 2/9 (22.2%) patients with diabetes mellitus. 7/9 patients (77.7%) of the herein investigated patients were highly symptomatic with a New York Heart Association functional class ≥III. Baseline left ventricular ejection fraction was preserved in 44.4% (4/9), moderately reduced in 33.3% (3/9), and severely reduced in 22.2% (2/9). One patient suffered from end-stage heart failure and s/p left ventricular assist device (LVAD) implantation. Detailed patient demographics are summarized in Table 2.

**Table 2:** Baseline data.

	**Study group (*n* = 9)**
Age, years	74.4 ± 7.1
Female gender, % (*n*)	88.8 (8)
BMI, kg/m^2^	25.1 ± 5.9
EuroSCORE II, %	5.5 ± 3.6
STS PROM Score, %	6.2 ± 3.0
Diabetes, % (*n*)	22.2 (2)
Arterial hypertension, % (*n*)	55.5 (5)
Previous stroke, % (*n*)	11.1 (1)
Coronary artery disease, % (*n*)	33.3 (3)
Previous sternotomy, % (*n*)	33.3 (3)
s/p LVAD implantation, % (*n*)	11.1 (1)
Extracardiac atheropathy^∞^, % (*n*)	11.1 (1)
Arrhythmia, % (*n*)	55.5 (5)
COPD^∞^> Gold II, % (*n*)	33.3 (3)
Creatinine, mg/dl	2.0 ± 2.3
NYHA ≥ III, % (*n*)	77.7 (7)
LVEF, >50%	44.4 (4)
LVEF, 50–30%	33.3 (3)
LVEF, >30%	22.2 (2)

*BMI, Body mass index; logEuroSCORE, Logistic european system for cardiac operative risk evaluation; STS-PROM, Society of thoracic surgeons predicted risk of mortality; LVAD, Left ventricular assist device; COPD, Chronic obstructive pulmonary disease; NYHA, New York Heart Association functional class; ∞ extracardiac atheropathy; ∞ COPD according to EuroSCORE definitions*.

### Periprocedural Data

Procedure time, fluoroscopy time, and volume of contrast agent used were 70.9 ± 32.4 min, 23.2 ± 13.8 min, and 212.3 ± 105.8 ml, respectively. The initial positioning of the THV differed from the standard procedure for AS. The THV was positioned ~1 mm higher in the aortic annulus compared to interventions for AS. For final deployment (step two), rapid pacing was used for stable THV release. For implantation in the LVAD patient, LVAD flow was reduced prior to valve deployment.

The majority of the patients was treated under local anesthesia and/or analgosedation (8/9, 88.8%). Detailed periprocedural data are summarized in Table 3.

**Table 3:** Periprocedural data.

	**Study group (*n* = 9)**
Severe aortic regurgitation, % (*n*)	100 (9)
Baseline peak gradient, mmHg	21.3 ± 12.2
Baseline mean gradient, mmHg	9.9 ± 5.7
Invasive pre-implant peak gradient, mmHg	4.4 ± 3.5
Invasive pre-implant mean gradient, mmHg	9.4 ± 7.4
Acurate neo, % (*n*)	11.1 (1)
Acurate neo 2, % (*n*)	88.9 (8)
Procedure time, min	70.9 ± 32.4
Fluoroscopy time, min	23.2 ± 13.8
Contrast agent, ml	212.3 ± 105.8
Predilatation, % (*n*)	0 (0)
Postdilatation, % (*n*)	0 (0)
**Anesthesia, % (** ***n*** **)**
General anesthesia	11.1 (1)
Local anesthesia/conscious sedation	88.8 (8)
Invasive post-implant peak gradient, mmHg	2.3 ± 2.7
Invasive post-implant mean gradient, mmHg	11.7 ± 7.1

### Clinical and Echocardiographic Outcome Data

All-cause 30-day mortality was 0% (0/9). Device success and early safety were 100% (0/9) and 77.7% (7/9), the latter due to two cases of acute kidney. The VARC-2 adjudicated clinical endpoints—stroke, myocardial infarction, or access site complication—did not occur. No postprocedural conduction disturbance or PPM implantation was observed. Intensive care unit and hospital stay were 1.7 ± 1.1 and 12.9 ± 8.8 days, respectively. Prolonged hospital stay was due to extensive preoperative diagnostic work-up because of planned off-label procedures. Echocardiography at 30 days revealed transvalvular peak/mean pressure gradients of 15.3 ± 12.3/7.2 ± 5.5 mmHg. PVL was ≤ trace in eight patients (77.7%, 7/9) and mild in two patients (22.2%, 2/9).

For detailed outcome parameter see Table 4.

**Table 4:** Clinical outcome and echocardiographic results at 30 days.

	**Study group (*n* = 9)**
All-cause mortality (30 days), % (*n*)	0 (0)
Stroke (any), % (*n*)	0 (0)
Myocardial infarction, % (*n*)	0 (0)
Bleeding (major/life threatening), % (*n*)	0 (0)
Access site complications (major), % (*n*)	0.0 (0)
Acute kidney injury (AKIN^*^ 2, 3), % (*n*)	22.2 (2)
PPM implantation, % (*n*)	0 (0)
Device success^†^, % (*n*)	100 (9)
Early safety^‡^, % (*n*)	77.7 (7)
Intensive care unit stay, days	1.7 ± 1.1
In hospital stay, days	12.9 ± 8.8
Peak gradient, mmHg	15.3 ± 12.3
Mean gradient, mmHg	7.2 ± 5.5
Mild PVL, % (*n*)	22.2 (2)
PVL > mild, % (*n*)	0 (0)

*PPM, Permanent pacemaker; PVL, Paravalvular leakage;*

* *AKIN, Acute Kidney Injury Network; VARC-2 definitions:*

† *Device success: absence of procedural mortality, correct positioning of a single prosthetic heart valve into the proper anatomical position, intended performance of the prosthetic heart valve (no prosthesis-patient mismatch and mean aortic valve gradient < 20 mmHg or peak velocity < 3 m/s and no moderate or severe prosthetic valve regurgitation)*,

‡ *Early safety at 30 days: all-cause mortality (at 30 days), all stroke (disabling and non-disabling), life-threatening bleeding, acute kidney injury stage 2 or 3 (including renal replacement therapy), coronary artery obstruction requiring intervention, major vascular complication, valve-related dysfunction requiring repeat procedure (Balloon aortic valvuloplasty, TAVI, or SAVR)*.

## Discussion

### Main Findings

Main findings of the herein conducted series using the Acurate neo or neo2 THV in TF TAVI for treatment of pure non-calcified AR are (I) the utilized device presents encouraging results in treatment of AR in patients not eligible for surgery, (II) clinical and echocardiographic results with no documented device migration/embolization and no PVL > mild suggest advantages of the particular design features of the THV for treatment of AR and (III) device success rate suggests that the herein recommended valve sizing algorithm may be especially appropriate for the Acurate neo 2 valve for this special subset of patients.

Over the last years, an increasing number of TAVI procedures for treatment of AR has been documented. Since prevalence of AR increases with age and physicians gather more experience with interventional treatment of this specific subset of patients, this trend is expected to continue. However, TAVI for AR represents certain pre- and intraprocedural challenges which are reflected by documented learning curves and uncertainty regarding adequate valve selection and sizing algorithms (20). For the most frequently used THV systems for treatment of AR varying clinical outcomes and valve sizing algorithms are described. A review of 31 published manuscripts by Yousef et al. (21) evaluating a variety of THV (CoreValve, JenaValve, Direct Flow, Acurate TA, J-Valve, Sapien, Lotus) showed unfavorable clinical outcomes with high 30-day mortality (9.6%), high intraprocedural need for a second THV (11.3%), high PPM rate (10.7%), and a high incidence of more than moderate PVL (17.7%). Although different valve types were combined in this review, oversizing was ≤ 10% in two thirds (66.4%) of cases (21). However, with latest generation devices and increased experience improved outcomes were documented over the last few years. Registry data shows that the most frequently used THV for treatment of AR is the SE CoreValve Evolut/EvolutR (14). Although documented outcomes with this THV generation are significantly improved compared to the initial CoreValve system, mortality, PPM, and residual significant PVL rates are still remarkable with 9, 20, and 6.2% respectively (12). A series of 24 AR patients who received the Acurate neo THV achieved a device success rate of 87.5%, with moderate PVL in two patients, both when valve oversizing was >10%. Furthermore, mortality was 4.1% and PPM rate 21.1% (13). These reports and our herein described data, although comprising only a small series of patients, suggest that especially the Acurate neo2 THV may be particularly suitable for treatment of AR. Nevertheless, those cited previous reports presented significant higher rates of residual PVL and need for a second THV as compared to implantation of this THV for treatment of AS. Reasons for absence of valve migration/embolization and no PVL > mild in our series may be a combination of a modified sizing algorithm with an oversizing ratio of >10% in the majority of patients, the positioning ~1 mm higher in the aortic annulus than in interventions for AS, the x-shaped stent frame preventing distal or proximal migration as well as extensive user experience with this type of THV. However, it has to be emphasized that the Acurate neo THV presented unfavorable results regarding residual significant PVL compared to a latest generation BE THV and inferiority regarding mortality and residual significant PVL compared to a latest generation SE THV in randomized controlled trials investigating TAVI in patients with AS (22, 23). Why these outcomes differ from the herein seen hemodynamic results remains speculative but may be founded in the valve sizing algorithm and the high implantation height. Reasons for the two cases of postoperative acute kidney injury remain speculative, but are highly likely attributable to preoperative existing reduced kidney function as reflected by preoperative creatinine values and preoperative reduced left ventricular function in the majority of the herein investigated patients.

Of note, in this AR patient cohort, the second deployment step was conducted under RVP to avoid THV dislocation. In one patient with status post LVAD implantation, LVAD flow was briefly reduced to a minimum during device deployment as an additional measure to avoid proximal embolization. The herein described more pronounced oversizing algorithm did not lead to conduction disturbances or postprocedural PPM implantation. This suggests that the documented high PPM rates with other THV systems may rather result from deep implantation height rather than oversizing alone. However, current THV systems are still used off-label and anchoring in non-calcified aortic annuli carries a certain risk for device migration. Therefore, for ideal results and patient safety, THV systems with modified anchoring mechanisms, like the only recently approved JenaValve with clipping of aortic valve cusps, might be advantageous (10).

These results will have to be confirmed in larger patient numbers for further clinical evaluation.

### Study Limitations

Limitations are inherent in a single-center study design with limited patient numbers: patients were not randomized to a specific treatment or THV, therefore patient preselection with hidden confounders may apply. Furthermore, this is a purely descriptive study and conclusions regarding feasibility and especially regarding long-term safety should be drawn with caution.

## Conclusions

In this limited series of TAVI using the Acurate neo THV for pure non-calcified AR, encouraging results were demonstrated. Thirty-day mortality as well as PPM implantation and significant PVL rates were 0% in this high-risk patient cohort. Whether these auspicious results are applicable in larger patient cohorts has yet to be confirmed since PPM and PVL rates differ significantly compared to AS patients provided with this particular THV.

## Data Availability Statement

The raw data supporting the conclusions of this article will be made available by the authors, without undue reservation.

## Ethics Statement

The studies involving human participants were reviewed and approved by UKE Ethics Committee. The patients/participants provided their written informed consent to participate in this study.

## Author Contributions

YS made substantial contributions to the conception and design of the work, the acquisition, analysis, interpretation of data for the work, and wrote the manuscript. OB made substantial contributions for the acquisition and analysis of the data. MS, AS, NS, SP, DW, SB, and HR were revising it critically for important intellectual content and made final approval of the version to be published. LC made substantial contributions to the conception and design of the work and the acquisition, analysis, interpretation of data for the work, and he was revising it critically for important intellectual content and made final approval of the version to be published. All authors contributed to the article and approved the submitted version.

## Conflict of Interest

MS reports lecture fees and travel expenses from Boston Scientific. LC is advisory board member for Boston Scientific. The remaining authors declare that the research was conducted in the absence of any commercial or financial relationships that could be construed as a potential conflict of interest.

## Publisher's Note

All claims expressed in this article are solely those of the authors and do not necessarily represent those of their affiliated organizations, or those of the publisher, the editors and the reviewers. Any product that may be evaluated in this article, or claim that may be made by its manufacturer, is not guaranteed or endorsed by the publisher.

## Abbreviations

AR: Aortic regurgitation
AS: Aortic stenosis
BE: Balloon-expandable
LVAD: Left ventricular assist device
PPM: Permanent pacemaker
PVL: Paravalvular leakage
RVP: Rapid ventricular pacing
SAVR: Surgical aortic valve replacement
SE: Self-expandable
TAVI: Transcatheter aortic valve implantation
TEE: Transesophageal echocardiography
TF: Transfemoral
THV: Transcatheter heart valve.
